# P08 Appearance matters: a case of adult-onset Parry-Romberg syndrome with linear scleroderma en coup de sabre and neurological involvement

**DOI:** 10.1093/rap/rkad070.029

**Published:** 2023-09-27

**Authors:** Zoe Syrimi, Zahirah Solim, Kiran Putchakayala

**Affiliations:** Mid Cheshire Hospitals NHS Foundation Trust, Crewe, United Kingdom; Mid Cheshire Hospitals NHS Foundation Trust, Crewe, United Kingdom; Mid Cheshire Hospitals NHS Foundation Trust, Crewe, United Kingdom

## Abstract

**Introduction:**

We describe the case of an adult patient who initially presented with neurological symptoms. Investigations were suggestive of a neuroinflammatory disorder but the diagnosis was unclear. It was only as time passed that the cutaneous signs and symptoms of Parry-Romberg syndrome (PRS) and linear scleroderma en coup de sabre (LSCS) became apparent to clinicians as the underlying cause for her presentation.

**Case description:**

This female patient, with no past medical history, presented in 2015 when she was 43 years old. She reported a one-day history of malaise and lethargy, left-sided headache, numbness affecting the right face and right arm and dysphasia. Her symptoms gradually improved over the following few days. An MRI brain showed extensive T2 hyperintensity in the right frontal and frontoparietal regions. Cerebrospinal fluid (CSF) showed a lymphocytosis and oligoclonal bands, which were present in the CSF only. A repeat MRI brain a few weeks later showed slightly less extensive changes. This was treated by neurologists as a neuroinflammatory disorder with intravenous methylprednisolone pulses. At follow-up, she mentioned that she had noticed a change in her appearance, with the right side of her face becoming increasingly asymmetrical over the last few years. Neurologists were puzzled regarding the diagnosis, but given that she was almost asymptomatic at review, they decided to monitor her with serial MRI scans. There were no further neurological episodes and no interval changes on repeat scans. In 2020, she was referred to ophthalmology, as she had noticed that her right eye had become increasingly sunken. MRI of the orbits and sinuses was reported to show enophthalmos of no obvious cause. Around the same time, she was also referred to dermatology for an enlarging patch of alopecia over the right frontoparietal scalp. Autoimmune screening blood tests including ANA, ENA, dsDNA and centromere were negative. Scalp biopsy was inconclusive but was most in keeping with old alopecia areata. She was treated with intralesional steroids and had a partial response. It was only by 2021 that PRS with LSCS was recognised as the cause of her symptoms. We can be confident that her neuroinflammation is an extracutaneous manifestation, which occurred early on when cutaneous signs were subtle.

**Discussion:**

PRS is a rare, acquired disorder characterised by progressive unilateral atrophy of facial skin, soft tissue, muscles, and underlying bony structures. LSCS is a subtype of linear scleroderma that occurs on the forehead or frontoparietal scalp. PRS and LSCS commonly coexist, and it is debated whether they are on a spectrum of the same disease. Both usually occur in childhood or adolescence, and adult-onset disease is very rare. The diagnoses are clinical and there are no specific diagnostic tests. Both are associated with neurological involvement, including seizures, headaches, focal neurological deficits and neuropsychiatric changes. Usually, the cutaneous lesion develops months to years before the onset of neurological symptoms. Our patient had noticed subtle changes in her appearance at least five years before her presentation in 2015. Neuroimaging abnormalities are predominately ipsilateral to the cutaneous lesions but may rarely be contralateral.

**Key learning points:**

Given that adult-onset PRS and LSCS are very rare, there may be a lack of awareness of the conditions amongst adult physicians. This can lead to diagnostic delay. This case highlights that PRS and LSCS are not just conditions of the skin and connective tissues and that neurological involvement is a frequent extracutaneous manifestation. This case is also a reminder of the importance of a thorough examination of the skin, especially when the diagnosis is unclear.

